# Setting the Stage for the Next Great Vaccine Success Story

**DOI:** 10.1128/mbio.00457-22

**Published:** 2022-06-06

**Authors:** Megan Culler Freeman

**Affiliations:** a University of Pittsburgh Medical Center, Pittsburgh, Pennsylvania, USA

**Keywords:** DNA cross-linking antibodies, nonpolio enterovirus, poliovirus, serology, vaccination

## Abstract

In this Commentary, the article by Rosenfeld et al. “Cross-Reactive Antibody Responses against Nonpoliovirus Enteroviruses” is put into context of the historic poliovirus epidemics and resultant vaccination success story as it compares to the current state of acute flaccid myelitis; the relationship to nonpoliovirus enteroviruses (EVs), in particular EV-D68 and EV-A71; and the potential for successful vaccination strategies. The discovery of cross-protective antibody neutralization among polio and nonpolio enteroviruses, specifically EV-D68, opens future questions about EV-D68 vaccination strategies, circulation patterns of nonpolio enteroviruses, and the interpretation of EV-D68 serostudies.

## COMMENTARY

Nearly 70 years ago, in February of 1954, a group of elementary school children were the first recipients of the Salk poliovirus vaccine down the street from the building where this article was written in Pittsburgh, Pennsylvania. The Salk inactivated poliovirus vaccine (IPV) contained all three types of poliovirus, types 1 to 3 (1). Poliomyelitis has been described since antiquity; however, epidemics of polio became increasingly severe in the early 1900s, likely due to increased sanitation and decreased repetitive exposure. Over 21,000 cases of paralytic polio cases were reported in the United States in 1952, most of them in children. Parents feared letting children gather in the summers when infections peaked, and pools and amusement parks were closed to halt spread. Public health interventions, such as travel bans and quarantines, were the only tools available to prevent illness. The elimination of poliovirus and resultant poliomyelitis in the United States and most of the world is one of the great success stories of vaccination. Global poliovirus cases have decreased over 99.9% since vaccination became widely available. Only a few countries with native poliovirus circulation remain before global eradication of this illness can be achieved.

Acute flaccid myelitis (AFM) has recently emerged as a poliomyelitis-like syndrome temporally associated with the summer-autumn circulation of nonpoliovirus enteroviruses (NPEVs), specifically enterovirus D68 (EV-D68) and enterovirus A71 (EV-A71). Outbreaks of EV-D68 as well as AFM in the United States peaked in 2014, 2016, and 2018, affecting hundreds of patients. Ninety percent of AFM cases are in children, with a median age of onset of 5 years. Another AFM peak was expected in the year 2020; however, the circulation of SARS-CoV-2 as well as nonpharmaceutical interventions of masking and distancing changed the circulation patterns of multiple respiratory viruses, including Respiratory Syncytial Virus (RSV), influenza, and EV-D68 (2). National surveillance noted fewer detections of EV-D68 and of AFM than expected during the year 2020. While some of the seasonality is thought to be related to weather patterns, the reason why poliovirus, as well as NPEV, have peak and trough years is not known, although one study suggested that population levels of serum antibody correlated with periodic EV epidemics (3, 4). It is also not known why AFM mostly affects children.

The degree to which EV-D68 has circulated historically has been challenging to ascertain. It was discovered in 1962 as a cause of viral pneumonia. While EV-D68 was recognized as a reemergent pathogen in 2012 as a cause of severe respiratory disease, molecular diagnostic techniques were not widespread. Most commercially available PCR-based multiplex diagnostic assays for upper respiratory viruses combine and report rhinoviruses and EV from a single target, such that further deconvolution is required to define an EV-D68 infection based on available clinical data. This strategy is how outbreaks were discerned in 2014 and 2016. Since 2017, the CDC has included EV-D68 in its active, prospective surveillance program (5). In 2018, this group found that pediatric patients with EV-D68 infections had a median age of 2.9 years, although this was higher in subsequent years (7.3 years in 2019 and 5.3 years in 2020, albeit with fewer detections overall) (6). After the association between EV-D68 and AFM was made, serologic studies of certain geographic distributions or of intravenous immunoglobulin (IVIG) were used to assess retrospective circulation.

The recent *mBio* report by Rosenfeld et al. (7), however, highlights that these serologic studies may be difficult to interpret due to a high degree of cross-reactivity of human antibodies generated after NPEV infections, as well as by routine poliovirus vaccination. Rosenfeld’s group investigated the potential for cross-reactive binding and cross-neutralization of antibodies generated against multiple EVs from species A to D against others. They discovered that EVs generate extensive cross-reactivity among the viral family that is not able to be predicted from genetic analysis alone.

They began by evaluating sera from nine healthy adults not known to have previously had EV-A71 or EV-D68 infections for the capacity of their antibodies to neutralize multiple different viruses. The strongest positive antibody response was to poliovirus, presumably due to vaccination, followed by lower *in vitro* neutralizing titers against EV-A71, and then two isolates of EV-D68, suggesting that neutralizing antibodies against multiple EV are present in healthy human sera. Because the specific infection history of these individuals was unknown, it is not possible to know whether these results were solely the product of polio vaccination or past infection with other NPEV. To specifically address how anti-EV antibodies could be cross-reactive, mice were vaccinated against a single EV, including multiple isolates of EV-D68, poliovirus type 1, EV-A71, EV-D94, and CVA-24v, with an adjuvanted and boosted strategy. The results showed that the most robust cross-reactivity was elicited between EV-D68, though not every isolate, and poliovirus. These studies were also replicated in a guinea pig model, suggesting that the findings were not species-specific. Similar to the results with healthy adult sera, mice who had been vaccinated against poliovirus could very effectively neutralize poliovirus but also had detectable neutralization titers against three of five isolates of EV-D68, as well as EV-A71, and a rhinovirus strain, HRV-A1A. Unfortunately, the cross-reactivity of EVD-68 vaccination-induced antibodies was not as high, with sera neutralizing only one of five EV-D68 isolates in addition to poliovirus.

The implications of this work are multiple. Given the number of NPEV genotypes (>110), genetically similar rhinovirus genotypes (>160), common circulation, and near-universal poliovirus vaccination, there is great potential for a robust and confounding cross-reactive antibody repertoire. This leads to reevaluation of conclusions drawn from previous serologic studies regarding NPEV circulation. The cross-reactivity between antibodies generated against poliovirus and EV-D68 neutralization is interesting and reminiscent of the recent discoveries of cross-reactive monoclonal antibodies that can neutralize multiple types of influenza (8) or that can neutralize both respiratory syncytial virus and human metapneumovirus (9, 10).

In the United States, since the year 2000, children receive four IPV doses in the primary childhood series. The first three doses occur in the first year of life, followed by a booster dose between four to 6 years of age. Greater than 90% of children develop protective antibodies after two doses, with at least 99% immunity after three doses. Studies of polio vaccine-induced antibodies over time have demonstrated that most children continue to have protective levels prior to their 4 to 6 year old booster. If antibodies produced as a result of poliovirus vaccination alone protected against the development of AFM, we should not be seeing this as a disease of young children. Given that poliovirus vaccination had been widely adopted in the United States by the time EV-D68 was discovered, it is possible that the contemporary genotypes of EV-D68 have developed a mechanism of immune evasion against the most commonly generated polyclonal anti-polio antibodies. Alternatively, perhaps a high degree of cross-neutralization in cell culture is not replicated when a patient encounters a high viral load *in vivo.*

The knowledge gained by this study may inform us about how an individual’s accumulated antibody repertoire, even acquired after poliovirus vaccination, may help protect against the development of AFM when exposed to EVs. Serostudies of children have suggested the capacity to neutralize EV-D68 reaches a nadir at 1 year of age but climbs to over 90% of participants by the teen years (11). Exposure to heterologous EVs over a period of several years may generate a collective humoral response that protects older individuals from developing AFM. Reinfection with respiratory viruses is the norm, as sterilizing immunity at the mucosal surface is difficult to maintain, a point both recently reiterated with SARS-CoV-2 and experimentally demonstrated for other respiratory viruses, such as RSV. While a person’s anti-EV antibody repertoire may not protect them from infection with circulating strains, humoral immunity previously generated by other circulating species may protect patients from developing severe diseases at secondary tissue sites, such as the central nervous system. The humoral immune response protects against neuroinvasive disease; this is how polio vaccine protects against poliomyelitis and is redemonstrated for NPEV in patients who have congenital humoral immune deficiencies. A well described complication of patients with X-linked agammaglobulinemia is chronic EV meningoencephalitis. Not only are antibodies required to protect against this illness, but they are also required to clear it, as these patients improve after being given IVIG.

This potentially cross-reactive or cross-protective antibody profile for EV may also help explain why EV-D68 seems to circulate in a biennial pattern. Circulation in 1 year could generate antibodies that provide mucosal surface protection for the following year, or it is possible that there is a partner NPEV or multiple NPEV that boost antibody response in the non-EV-D68 year and prevent circulation/infection in the off year. There is much that we do not understand about the ecology of circulating human respiratory viruses, again demonstrated by the altered circulation patterns of RSV, influenza, and others during the SARS-CoV-2 pandemic. How might the circulation of one NPEV affect the circulation of others? If this is the mechanism that prevented a large EV-D68 year in 2020 as well as a peak in AFM, then should we be prepared for a potentially even larger spike of EV-D68 and resultant AFM in the coming season due to more immunologically naive hosts?

The study by Rosenfeld et al. (7) also illustrates how challenging it may be to design an EV-D68 and/or EV-A71 vaccine that protects against AFM. For vaccination against poliovirus to be successful, all three types had to be included, as vaccination with one type did not protect from disease associated with the other two types. Unfortunately, there are more than three genotypes of EV-D68, and as shown in this work, vaccination with one strain was not sufficient to neutralize *in vitro* against all others. If we are to turn vaccination against NPEV and prevention of AFM into the next great vaccination success story, there is much work ahead to understand both the specific antibody responses required for protection and the immunologic interplay between poliovirus and NPEV.
